# Development of Advanced 3D-Printed Solid Dosage Pediatric Formulations for HIV Treatment

**DOI:** 10.3390/ph15040435

**Published:** 2022-03-31

**Authors:** Azizah M. Malebari, Aytug Kara, Ahdab N. Khayyat, Khadijah A. Mohammad, Dolores R. Serrano

**Affiliations:** 1 Department of Pharmaceutical Chemistry, College of Pharmacy, King Abdulaziz University, Jeddah 21589, Saudi Arabia; amelibary@kau.edu.sa (A.M.M.); ankhayyat@kau.edu.sa (A.N.K.); kmohammad@kau.edu.sa (K.A.M.); 2 Department of Pharmaceutics and Food Science, Facultad de Farmacia, Universidad Complutense de Madrid, 28040 Madrid, Spain; akara@ucm.es; 3 Instituto de Farmacia Industrial y Galénica, School of Pharmacy, Universidad Complutense de Madrid, 28040 Madrid, Spain

**Keywords:** pediatric formulation, ritonavir, lopinavir, minitablet, 3D printing, direct powder extrusion, HIV, FDM, HME

## Abstract

The combination of lopinavir/ritonavir remains one of the first-line therapies for the initial antiretroviral regimen in pediatric HIV-infected children. However, the implementation of this recommendation has faced many challenges due to cold-chain requirements, high alcohol content, and unpalatability for ritonavir-boosted lopinavir syrup. In addition, the administration of crushed tablets has shown a detriment for the oral bioavailability of both drugs. Therefore, there is a clinical need to develop safer and better formulations adapted to children’s needs. This work has demonstrated, for the first time, the feasibility of using direct powder extrusion 3D printing to manufacture personalized pediatric HIV dosage forms based on 6 mm spherical tablets. H-bonding between drugs and excipients (hydroxypropyl methylcellulose and polyethylene glycol) resulted in the formation of amorphous solid dispersions with a zero-order sustained release profile, opposite to the commercially available formulation Kaletra, which exhibited marked drug precipitation at the intestinal pH.

## 1. Introduction

3D printing technologies allow the fabrication of personalized medicines adapted to customers’ needs [1]. Opposite to the “one-size fits all” concept, 3D printing enables the manufacture of pharmaceutical dosage forms according to patient requirements, such as dose, release profile, color, texture, and size [2,3]. This is especially important in the pediatric population, bearing in mind its different needs compared to those of adults [4]. Dose adjustment is a key factor to ensure efficacy but with minimal adverse effects. Most oral medications for children rely on the use of liquid pharmaceutical dosage forms, which may result in drug stability issues [4]. On many occasions, solid dosage forms are also used, but commonly, they need to be titrated, which can lead to poor patient compliance and an inaccurate doses [5].

According to the World Health Organization (WHO), 1.7 million children aged 0–14 were living with human immunodeficiency virus (HIV) at the end of 2018; 160,000 children were newly infected, and 100,000 died of acquired immunodeficiency syndrome (AIDS)-related illnesses [6]. Early testing and treatment are essential to reduce HIV-related mortality and morbidity among children. However, only 52% of children with AIDS received antiretroviral therapy, and only half of them received optimal treatment regimens [6]. By far, the progress made in optimizing HIV therapies in adults is much greater than in the pediatric population, which continues to receive suboptimal treatment. Personalization of pediatric solid dosage forms plays a key role in the management of this complex disease. Despite WHO recommending ritonavir-boosted lopinavir-based antiretroviral regimens for infants and young children since 2013, implementation of this recommendation has faced many challenges due to cold-chain requirements and unpalatability for ritonavir-boosted lopinavir syrup [7].

As a start, 3D printing can overcome the issues derived from lacking personalized HIV medicines for children [8]. 3D printing is used in many biological fields, and currently, there is a wide range of 3D printing technologies to manufacture solid dosage forms [9,10]. However, more research is necessary to bring this innovative technique into clinical practice combining engineering, molecular chemistry, and pharmaceutical technology [11]. Fused deposition modeling (FDM) has been widely used in the fabrication of oral personalized medicines, including multiple drugs [12]. However, prior to the FDM printing, the manufacture of a drug-loaded filament by hot-melt extrusion (HME) is required. This limits its translation into clinical practice, as it increases the likelihood of drug degradation through the thermal effect, and the batch size for extrusion is relatively large, and the optimization of the formulation is complex [13,14]. The drug’s passive diffusion into industrially fabricated filaments can be an alternative. However, drug loading using this approach tends to be poor, usually below 1%. Several authors have reported drug loadings up to 3% when the process is optimized [15], but the pediatric dose for ritonavir-boosted lopinavir medicines requires greater drug loadings to ensure that a small solid dosage form is fabricated to be swallowed easily. Different approaches have been utilized to produce ritonavir-boosted lopinavir pediatric formulations, such as granules utilizing in situ self-assembly nanoparticles [16] or fast-dissolving tablets [17]. However, the manufacture of these formulations in clinical settings is complicated.

Direct powder extrusion (DPE) has emerged as a novel 3D printing approach in which a powder mixture is loaded directly into the 3D printer, and upon heating, the API-excipient mixture is deposited according to the geometrical designed [18]. This technique overcomes limitations around drug loading that usually occur with conventional HME, as the latter needs a greater quantity of excipients to prevent fabricating too-brittle or too-flexible filaments unable to be loaded into an FDM printer [14]. Compared to semisolid extrusion (SSE), in which the starting material is a semi-solid or semi-molten material, DPE has the advantage of no solvents being required for printing, and, therefore, 3D-printed structures are harder, being less friable and free of solvents toxic to humans [18]. Therefore, DPE can simplify 3D printing into a more cost- and time-effective single-step manufacturing process [19,20].

The hypothesis underpinning this work is that direct powder extrusion can be utilized for manufacturing minitablets of ritonavir and lopinavir adjusted to children’s needs. Their small size will enhance children’s compliance, and at the same time, their solid state will enhance drug oral bioavailability. Previously, we demonstrated that spherical minitablets of nifedipine can be successfully manufactured using FDM coupled with HME, resulting in a controlled release formulation [13]. In this work, we have explored the potential of direct powder extrusion compared to FDM to fabricate spherical minitablets as an easier technique to implement in hospital settings. The 3D-printed spherical minitablets of ritonavir and lopinavir have been designed and fabricated, and their physicochemical characteristics have been investigated and compared with Kaletra, the commercially available solid dosage form containing both ritonavir and lopinavir.

## 2. Results

### 2.1. Manufacture of 3D-Printed Minitablets

The spherical minitablets were printed according to the dimensions of the CAD file. Initially, minitablets were fabricated by hot-melt extrusion, followed by fused deposition modeling, but this process led to a significant drug degradation (>30%) at 120 °C, the temperature required to obtain printable filaments. For this reason, FDM was replaced with direct powder extrusion, in which the temperature was reduced to 80 °C. The ritonavir minitablets were slightly smaller in weight and diameter compared to the lopinavir ones (Table 1). However, no statistical significance was observed between the two tablets.

The drug content at the conditions utilized for 3D printing was maintained above 90%, meeting the quality specifications. In all the parameters evaluated, weight, diameter, density, and drug content, the coefficient of variation was lower than 15%, showing a good reproducibility of the 3D printing process meeting Pharmacopeia specifications. Before direct powder extrusion, both minitablets were fabricated through hot-melt extrusion, followed by fused deposition modeling 3D (FDM) printing (data not shown). The temperature required to obtain suitable filaments from the hot melt extruder was about 120 °C, which elicited a significant degradation of the drug content, making it unsuitable for its translation into clinical practice. For this reason, FDM was replaced with direct powder extrusion, and a mixture of powders was directly used for printing. The temperature was reduced to 80 °C, and the residence time inside the heating barrel of the extruder was much shorter (<10 min), which allowed an enhanced control of the printing process and avoided drug degradation. The printing time for each minitablet was 6 min, and thereafter, full therapy for a one-month treatment could be fabricated in 3 h.

### 2.2. Solid State Characterization

#### 2.2.1. Morphology

Even though five different types of polymorphs have been identified for ritonavir, form I and II are the most common ones characterized by a needle and rod-like crystal habit, respectively [23]. Polymorph I was the first discovered, but polymorph II is the thermodynamically more stable but much less water-soluble crystalline form [23]. Additionally, the other three polymorphs have been characterized. Polymorph III is a crystalline solvate that converts to polymorph V, a hydrated phase upon exposure to moisture that turns spontaneously into needle-like polymorph I crystals, and polymorph IV is an unsolvated, metastable polymorph of ritonavir [23,24].

**Figure 1:**
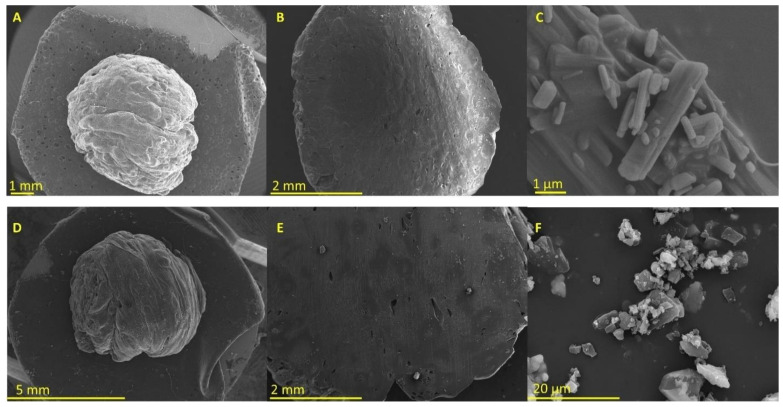
Micrographs were obtained from 3D-printed minitablets and unprocessed drugs. Key: (**A**) ritonavir 3D printed tablet, (**B**) ritonavir 3D-printed tablet cut in half, (**C**) unprocessed ritonavir, (**D**) lopinavir 3D-printed tablet, (**E**) lopinavir 3D-printed tablet cut in half, and (**F**) unprocessed lopinavir.

In Figure 1C, the crystal habit of unprocessed ritonavir is exhibiting a rod-like shape corresponding to polymorph II [25]. In contrast, the 3D-printed minitablet containing ritonavir shows a homogenous surface (Figure 1A) and core (Figure 1B) without the presence of crystals deposited on the surface.

The layers were not well defined, which can be attributed to the lower temperature utilized for printing to avoid drug degradation. In Figure 1F, the crystal habit of the unprocessed lopinavir is exhibiting a plate-like shape. Similar to the ritonavir minitablets, those containing lopinavir also showed a smooth surface. However, few crystals were found in the core of the tablet when these were cut in half (Figure 1E).

#### 2.2.2. Differential Scanning Calorimetry

**Figure 2:**
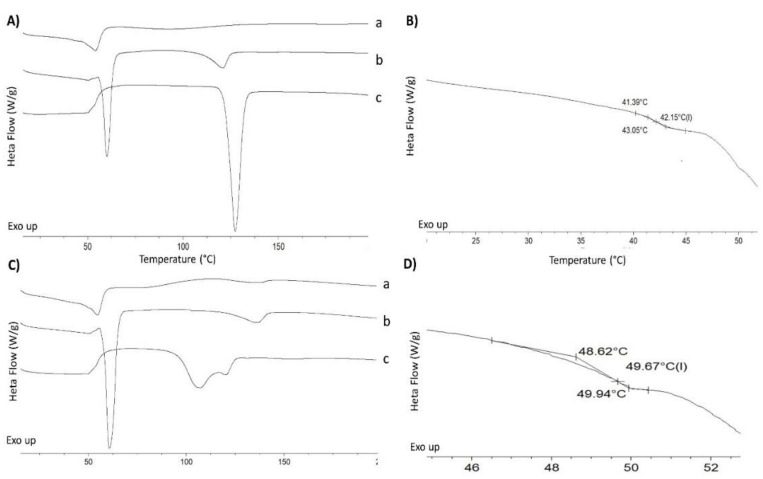
DSC thermograms of 3D-printed minitablets, physical mixtures, and unprocessed materials. Letters (**A**,**B**), ritonavir formulations and (**C**,**D**), lopinavir formulations. In graphs (**B**,**D**), the section corresponding to the glass transition temperature from the 3D-printed minitablet formulation has been enlarged. Key: 3D-printed minitablet (a), physical mixture (b), unprocessed drug (c).

Unprocessed ritonavir had a sharp melting point, attributed to its crystalline nature with an onset melting temperature of 122.4 ± 0.5 °C and enthalpy of fusion of 89.4 ± 1.4 J/g (Figure 2A). This is attributed to polymorph II of ritonavir [23]. The physical mixture of ritonavir with PEG 4000 and HMPCAS showed a depression in the melting point of the ritonavir at 111.4 ± 0.9 °C and the presence of the melting of PEG4000 at 57.5 ± 0.8 °C (Figure 2A). The thermogram of the 3D-printed minitablet showed the absence of the melting point attributed to ritonavir and depression of the onset melting of PEG (47.8 ± 1.1 °C) with a reduced enthalpy (Figure 2A). A clear glass transition was observed at 42.1 ± 0.4 °C (Figure 2B). As previously described, amorphous ritonavir does not crystallize spontaneously when heated above the Tg [26]. Similar behavior is observed with the ritonavir 3D-printed minitablets. Ritonavir can be considered an excellent glass former, bearing in mind that the ratio between its Tg/Tm is 0.82, and, thus, it can be expected that the amorphous solid dispersion tends to be physically stable compared to other drugs with a greater tendency for crystallization, such as griseofulvin. In addition, the formation of solid solutions between PEG and ritonavir has been described, in which the crystal lattice of ritonavir is destroyed, accompanied by partial destruction of the lattice of the PEG, which results in an improved release in aqueous media [27]. The PEG acts as a plasticizer, bringing down the Tg of amorphous ritonavir from 51 to 42 °C in the 3D-printed minitablets [26].

In the case of lopinavir, the melting point was not as sharp as for the ritonavir. A double endothermic peak was observed at 97.4 °C and 117.8 °C, which can be attributed to a partially amorphous raw material (Figure 2C). This behavior has also been demonstrated in crystals obtained from ethyl acetate [28]. Surprisingly, the physical mixture showed a sharp endothermic peak at 57.9 ± 0.4 °C, attributed to the PEG 4000, and a second broad enthalpic event at 123.1 ± 0.5 °C, which is attributed to the lopinavir, taking into account that the second excipient, the HPMCAS, is an amorphous polymer that does not exhibit any endothermic event at this temperature (Figure 2C). The 3D-printed minitablet showed a glass transition at 49.3 ± 0.4 °C and a depression of the melting peak observed in the physical mixture (49.6 °C and 118.6 °C, respectively) (Figure 2C,D). Similar to the ritonavir formulation, it is expected that PEG also act as a plasticizer, reducing the Tg of the lopinavir from 75 to 50 °C in the 3D-printed minitablets [28].

#### 2.2.3. X-ray Powder Diffraction

**Figure 3:**
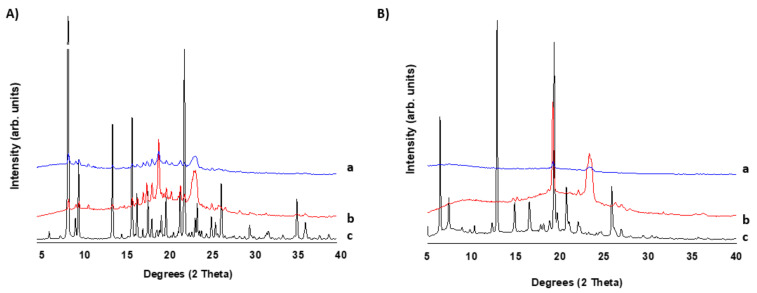
The pXRD diffractograms of 3D-printed minitablets, physical mixtures, and unprocessed materials. (**A**) Ritonavir, (**B**) lopinavir. Key: 3D-printed minitablet (a), physical mixture (b), and unprocessed drug (c).

In Figure 3, the pXRD of 3D-printed tablets is compared to those of unprocessed drugs and physical mixtures. Ritonavir exhibited Bragg peaks attributed to the polymorph II with an orthorhombic crystal lattice (Figure 3A) [29]. The physical mixture is characterized by the presence of Bragg peaks attributed to the crystalline components of the tablet, such as ritonavir and PEG. Due to the small fraction of magnesium stearate in the mixture, its Bragg peaks are not observed in the diffractogram. The 3D printed minitablets also showed Bragg peaks but of reduced intensity compared to those obtained in the diffractogram of the physical mixture. This indicates that traces of crystalline ritonavir are found in the minitablets even after the extrusion process that takes place during direct powder extrusion. The pXRD pattern for the unprocessed lopinavir corresponds to the crystal habit reported for lopinavir recrystallized from ethyl acetate [28]. The physical mixture showed Bragg peaks attributed to those of lopinavir and PEG. The 3D printed lopinavir minitablet showed peaks at the same 2 theta degrees but of lesser intensity than those observed in the physical mixture, which indicates the existence of a combination of crystalline and amorphous domains after the 3D printing process.

#### 2.2.4. Fourier Transformed InfraRed Spectroscopy

**Figure 4:**
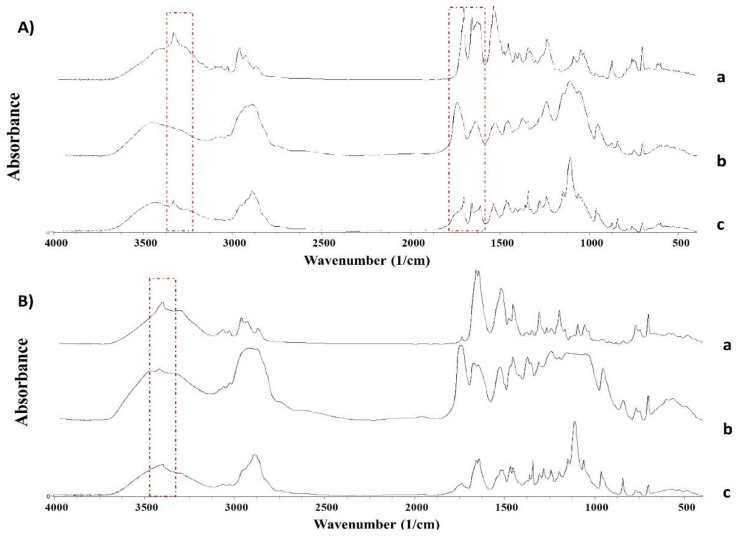
FTIR spectra of 3D-printed minitablets, physical mixtures, and unprocessed materials. (**A**) Ritonavir, (**B**) lopinavir. Key: unprocessed drug (a), 3D-printed minitablet (b), physical mixture (c).

The FTIR spectra show marked differences between the unprocessed drugs and the 3D-printed minitablets (Figure 4). The FTIR spectra between unprocessed ritonavir and 3D-printed minitablets showed a clear shift in the peaks attributed to the C=O amide peak from 1662 to 1637 cm^−1^; the C=O ester bond from 1706 cm^−1^ to 1737 cm^−1^; and the N-H stretch at 3328 cm^−1^, which disappeared in the minitablet (Figure 4A). Additionally, broader peaks were observed in the ritonavir minitablets, which can be attributed to the dominant amorphous nature of the system formed during 3D printing. In the case of lopinavir 3D-printed minitablets, the peak shifts compared to unprocessed lopinavir were only evident in the N-H stretch at 3403 cm^−1^. Broader peaks were also observed due to the partially amorphous nature of lopinavir in the 3D-printed minitablet.

### 2.3. Dissolution Profile

**Figure 5:**
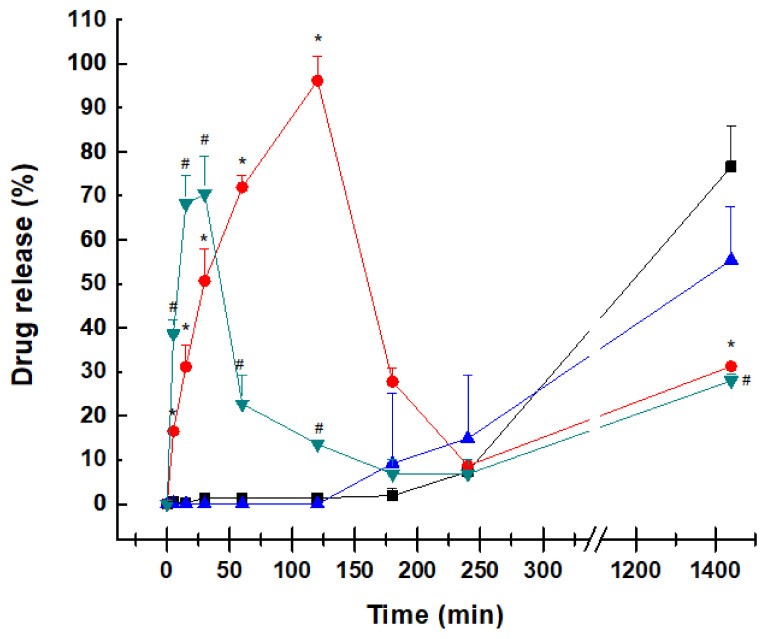
Dissolution release profile from 3D-printed minitablets containing ritonavir or lopinavir and the commercially available formulation (Kaletra). Key: ritonavir 3D printed minitablets (-■-), lopinavir 3D printed minitablets (-▲-), ritonavir from Kaletra tablets (-●-), and lopinavir from Kaletra tablets (-▼-). * Statistical differences (*p* < 0.05) between ritonavir from Kaletra tablets and 3D-printed minitablets. ^#^ Statistical differences (*p* < 0.05) between lopinavir from Kaletra tablets and 3D-printed minitablets.

The release profile of both 3D-printed minitablets was significantly different from the marketed formulation Kaletra. Kaletra is a coated tablet with an immediate release of both active ingredients, as shown in Figure 5. The core contains copovidone, sorbitan laurate, anhydrous colloidal silica, and sodium stearyl fumarate, resulting in an amorphous solid dispersion to enhance the solubility of both drugs and improve their oral bioavailability in the gastrointestinal tract [30,31]. The dissolution profile of both active ingredients is characterized for an immediate release in acid media, where ritonavir and lopinavir have greater solubility, followed by sharp precipitation at the intestinal pH. The release in acid media follows a first-order kinetic, with an R^2^ of 0.997 and 0.948 for ritonavir and lopinavir, respectively. The spring and parachute effect, commonly associated with supersaturation followed by retarding precipitation of the co-formulated drugs within the amorphous solid dispersion, is not observed in the dissolution profile of Kaletra. Even in acid pH, lopinavir shows a reduced release from the tablet compared to ritonavir, which can be due to its poorer solubility at acid pH (4.05 µg/mL at 37 °C pH 2) compared to ritonavir (400 µg/mL) [32], which can lead to a detriment in its oral bioavailability.

Contrary to the dissolution profile of Kaletra, the 3D-printed minitablets showed a negligible dissolution of ritonavir and lopinavir at acid pH, followed by a zero-order release at pH 6.8 over 24 h (R^2^ > 0.99). The tablets did not disintegrate into smaller particles as dissolution occurred from erosion of the tablet surface. No drug precipitation was observed at any moment. HMPCAS was used to maintain the integrity of the minitablet in the acid environment and prevent drug recrystallization difficult to redissolve at the intestinal pH. Keeping both drugs in solution at the intestinal pH can be beneficial to enhancing their oral bioavailability in the gastrointestinal tract.

When compared to Kaletra, the drug dose within the 3D-printed formulations is significantly smaller, as the formulation was designed for pediatric use. The main advantage of the smaller dose is that pill size can be also kept small and thus be more easily swallowed, but also, dose adjustment is more flexible based on children’s requirements. However, this can affect the dissolution profile, bearing in mind the poor water solubility of lopinavir being beneficial for the 3D-printed minitablets. This is one of the limitations of the current dissolution study. Drug release studies from the 3D-printed minitablets were performed separately for ritonavir and lopinavir, which may also impact their overall solubility and dissolution profile. However, the aim was to test if the novel formulations could keep a sustained release profile at the intestinal pH controlled by matrix erosion rather than tablet disintegration into smaller fragments.

## 3. Discussion

The combination of lopinavir/ritonavir remains one of the first-line therapies for the initial antiretroviral regimen in pediatric HIV-infected children both in the USA and Europe, starting with children as young as 2 weeks old. Protease inhibitor regimens have several advantages, such as excellent virologic potency and a high barrier to drug resistance, as multiple mutations are required for a patient to develop resistance [33].

In this manuscript, the feasibility of using 3D printing to manufacture personalized pediatric HIV solid dosage forms for overcoming the challenges of metastable polymorphs and unpalatability of the ritonavir-boosted lopinavir syrup has been demonstrated for the first time. The direct powder extrusion 3D printing resulted in minitablets in which the ritonavir and lopinavir were mostly in the amorphous state, through H-bonding with the polymers utilized, resulting in an optimal dissolution profile. Considering that 3D-printed medicines will be extemporaneously dispensed as demanded by patients over a 30-day period, physicochemical drug stability should not be a major issue to face. The unpalatability issue is resolved by the solid state of the tablet, which is small enough to be easily swallowed by children, and active ingredients can be combined as prescribed by the clinicians. Apart from the unpleasant taste of the oral suspension, this liquid formulation has high alcohol content (42%), which can result in alcohol toxicity with overdose, especially in infants [34]. In the past, this problem was addressed by crushing the tablets and administering them to children. However, years later, it was demonstrated that the administration of crushed tablets containing ritonavir and lopinavir in children is not advisable, as the oral bioavailability is significantly reduced for both drugs in approximately 45–50% of cases [34].

From a manufacturing point of view, ritonavir garnered much attention when a previously unknown, thermodynamically more stable polymorph appeared unexpectedly, causing serious issues for the commercialized product, as well as the patients taking the drug. Initially, polymorph I was only known by Abbott Laboratories, the pharmaceutical company that first commercialized ritonavir [29]. To overcome insufficient oral bioavailability, ritonavir was formulated as a capsule filled with a hydroalcoholic solution that contained the drug fully dissolved. Two years after coming to the market, the formulation began failing dissolution specifications. After a thorough investigation, it was discovered that polymorph I precipitated in the formulation, transforming into polymorph II, the one thermodynamically more stable but 50% less soluble, which led to poor dissolution behavior and withdrawal of the capsules from the market [29]. A new amorphous solid dispersion was eventually launched years later. This highlights the importance of understanding the nature of polymorphic shifts, as well as drug formulation.

The oral bioavailability of both ritonavir and lopinavir is limited. The fraction of the absorbed dose of ritonavir is estimated to be greater than 60–80% based on the recovered amount of unchanged drug in the feces. However, oral bioavailability is highly affected in the presence of food with a 7% lower AUC (area under the concentration-time curve) [35]. Lopinavir has very low oral bioavailability (~25%) when administered alone, and, thus, it is co-administered with ritonavir to enhance its oral absorption, hinder drug metabolism, and allow for the attainment of therapeutic drug concentrations [36]. In in situ intestinal perfusion experiments, ritonavir has been shown to be able to increase 2.3-fold the lopinavir intestinal permeability. Approximately, the highest plasma concentration is achieved at 4 h for both drugs in the conventional formulation, indicating relatively low absorption from the gastrointestinal tract [30]. This low and highly variable oral absorption of both drug inhibitors is related to its poor aqueous solubility at the intestinal pH. Kaletra tablets are formulated as an amorphous solid dispersion with the aim of enhancing drug–water solubility. In dissolution studies, a supersaturated solution of both ritonavir and lopinavir was achieved, which was expected to follow Ostwald’s rule and triggered the precipitation of both drugs at basic pH. This can explain the limited fraction of dose absorbed. However, the dissolution profile of the minitablets followed a zero-order release, keeping both drugs in solution, even at the intestinal pH. This can be a substantial advantage compared to Kaletra, taking into account the enhanced solubilized drug fraction over prolonged periods and the variability of the gastrointestinal pH in children, which can result in a large oral bioavailability [37].

The 3D-printed minitablets of lopinavir have been previously manufactured by selective laser sintering with a disintegration time below 2 min and a fast dissolution in less than 30 min [38]. However, the dissolution profile was not investigated at the intestinal pH, which can trigger the precipitation of both drugs in the gut, hampering their oral bioavailability. In this work, the 3D-printed minitablets fabricated by direct powder extrusion combining HMPCAS with PEG 4000 were able to maintain in solution the fraction dissolved from both drugs despite their poor water solubility at the intestinal pH. The fabrication of each minitablet was relatively fast, and in less than 3 h, a 30-day treatment could be created in a hospital pharmacy.

Regarding the dose to be administered, the minitablet size and drug content were designed appropriately. Kaletra oral solution contains 80 mg of lopinavir and 20 mg of ritonavir per each ml of oral solution. To solubilize both drugs at this concentration, 42.4% (*v*/*v*) of ethanol, 15.3% (*p*/*v*) of propylene glycol, and a similar content of corn sugar with a high fructose content must be used. The use of minitablets that are easy to swallow can reduce the intake of these excipients that are not beneficial for the child. A dose of 16 mg and 4 mg of lopinavir and ritonavir, respectively, per kg of weight are recommended. The current tablets contain 25% drug (equivalent to 40 mg). This means that to treat HIV in a child of 10 kg, it is necessary to administer one minitablet of ritonavir and four minitablets of lopinavir. The exact dose required for the child can be easily adapted based on the diameter of the tablet or the number of tablets administered. Additionally, the release profile attempts to keep both drugs solubilized even at the intestinal pH, which can favor their oral absorption compared to the commercial formulation being a necessitated lesser dose. However, further pharmacokinetic studies are required to prove this point.

## 4. Materials and Methods

### 4.1. Materials

Lopinavir and ritonavir were purchased from Kemprotec (>95%, Carnforth, UK). PEG 4000 and magnesium stearate were purchased from Sigma–Aldrich (Madrid, Spain). Hypromellose Acetate Succinate (AQOAT-LG grade) was a gift from Shin-Etsu (Tokyo, Japan). Kaletra containing lopinavir (200 mg) and ritonavir (50 mg) was kindly supplied by the Ramon and Cajal Hospital (Madrid, Spain), which was bought from Abbvie farmaceutica S.L.U. Solvents were HPLC-grade and were purchased from Proquinorte (Madrid, Spain). Any other reagents were used without further purification.

### 4.2. Methods

#### 4.2.1. Geometrical Design

**Figure 6:**
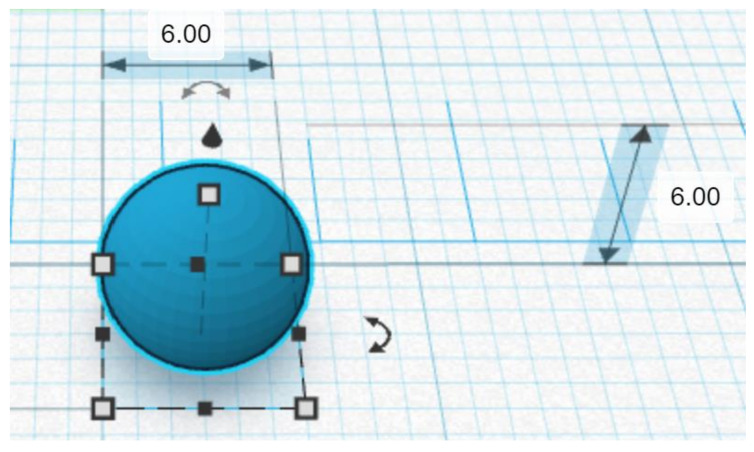
The geometric design of the solid minitablet. Lateral CAD view. Numerical values are expressed in millimeters.

A spherical minitablet geometry was designed using a computer-aided design (CAD) software (Tinkercad software v.1, Autodesk 2019, Barcelona, Spain) to create an STL file compatible with the Hyrel 3D printer software (Norcross, GA, USA) required to adjust the printing parameters (Figure 6). Minitablets were designed as spherical shapes with a diameter of 6 mm to fit within a size 0 hard capsule. The total surface area of each minitablet was calculated using Meshlab v.1.5.2. (Visual Computing Laboratory, CNR-ISTI, Pisa, Italy) to be 133.1 mm^2^.

#### 4.2.2. Formulation Development: DPE vs. FDM

A 20 g batch size was prepared containing 25% drug, either ritonavir or lopinavir (5 g), 51.75% HPMCAS (10.35 g), 22.5% PEG4000 (4.5 g), and 0.75% magnesium stearate (0.15 g). Ball milling (IKA Ultra-Turrax Tube Drive Disperser) was used to blend the powder mixture for 2 min at 6000 rpm, after which the mixture was sieved through a 0.35 mm screen before extrusion. The rationale of this ratio was previously investigated for the DPE of nifedipine [14]. A higher percentage of PEG4000 was required to bring the extrusion temperature below the drug degradation temperature.

#### 4.2.3. DPE Printing Settings

The 3D printer Hyrel 3D SR (Norcross, GA, USA) was used to print the minitablets. The powder was filled in a TAM-15 extruder with a 1 mm extrusion nozzle aperture. The platform temperature was set at 80 °C, and the printing temperature was set at 80 °C for all formulations. Ritonavir and lopinavir degradation were minimal at this temperature, which was verified by HPLC. Lastly, the print and travel speeds were set at 10 and 50 mm/s, respectively. Other relevant settings are summarized in Table 2 for all formulations.

#### 4.2.4. HME Coupled with FDM

HME of the mixture was performed with a Noztek touch single-screw extruder (Shoreham, UK) at 30 rpm. The temperature used during the extrusion was 120 °C. A 50 g batch size was prepared using the same composition as in Section 4.2.3. The extruded filaments were fed into a FlashForge Creator Pro (Zhejiang Flashforge 3D Technology Co., Zhejiang, China) FDM 3D printer, which was used to print the minitablets using the same settings described in Table 1.

#### 4.2.5. Mass and Content Uniformity

Minitablets (*n* = 10) obtained by direct powder extrusion were weighed and dissolved in methanol (50 mL), and drug content was quantified by HPLC (Section 4.2.8). The target drug loading for both drugs was 40 mg per tablet. The density of each tablet was calculated by dividing the weight of each tablet by the volume that occupies what is considered to be a perfect sphere. The dimensions of the minitablet were measured with a caliper (Cole Parmer, Fisher Scientific, Madrid, Spain). The coefficient of variation (CV) was calculated as the ratio of the standard deviation, and the average mean value of each parameter was multiplied by 100.

#### 4.2.6. Scanning Electron Microscopy (SEM)

A scanning electron microscope (JSM 6335F JEOL, Tokyo, Japan) was used at 5 kV after the samples were sputtered and coated with pure gold (Q150RS Metalizador QUORUM, UK) for 180 s. Micrographs were obtained from 100% fully printed minitablets and tablets printed only up to 50% to investigate the morphology of the core. Raw materials, including ritonavir and lopinavir powder, were also assessed.

#### 4.2.7. Solid-State Characterization

Solid-state characterization was performed on 3D-printed minitablets, unprocessed excipients, and active ingredients. Physical mixtures between drugs and excipients were prepared in an agar mortar and pestle and were also analyzed. Drug and excipients were weighed in the same ratio as contained in the 3D-printed minitablets. The powders were transferred into an agar mortar and pestle and were manually mixed before analysis. A thin slice (1–2 mm) of the 3D-printed filaments and minitablets was cut and analyzed to avoid grinding.

##### Fourier Transform Infrared (FTIR) Spectroscopy

FTIR analysis of ritonavir and lopinavir printed minitablets was carried out with a Nicolet Nexus 670–870 (Thermofisher, Madrid, Spain). A wavelength range from 400 to 4000 cm^−1^ was used. Each sample (1–3 mg) was mixed manually in an agate mortar and pestle with KBr (200 mg). The powder mixture was then compressed into compacts using a PerkinElmer hydraulic press set at a pressure of 10 tons for 10 min dwell time. Spectragryph (version 1.2.9, Oberstdorf, Germany) software was used for the interpretation of the spectra.

##### X-ray Powder Diffraction (pXRD)

Powder X-ray analysis was performed using a PANalytical diffractometer (X’ Pert, Malvern, Madrid, Spain) with Ni-filtered Cu Kα radiation (1.54 Å). The tube voltage and tube current used were 40 kV and 40 mA, respectively. The PXRD patterns were recorded (*n* = 3) from 5° to 40° on the 2 theta scale at a step scan rate of 0.05° per second at 25 °C.

##### Differential Scanning Calorimetry (DSC)

DSC scans were recorded on a QA-200 TA instrument (TA instruments, Elstree, UK) calorimeter using nitrogen as the purge gas. A section of the minitablets was cut, weighed (4–6 mg), and sealed in an open aluminum pan. The temperature range was set between 10 °C to 200 °C, and the heating rate was 10 °C/min. Calibration of the instrument was carried out using indium as standard. Glass transition temperatures reported (*n* = 3) were the midpoint of the transition.

#### 4.2.8. Dissolution Studies

Dissolution tests were performed in triplicate using a United States Pharmacopoeia (USP) apparatus 2 (ERWEKA DT 80, Heusenstamm, Germany) at 50 rpm (United States Pharmacopeia and National Formulary, USP38-NF33, Rockville, Maryland, USA). The employed dissolution media consisted of USP simulated gastric fluid (SGF) without enzymes (pH 1.2) and USP simulated intestinal fluid (SIF) without enzymes (pH 6.8), as described in the USP [22]. SGF (500 mL) was used during the first 2 h. SIF (400 mL) was added and kept for the remaining 22 h, and media were maintained at 37 ± 0.5 °C. NaOH (30% *v*/*v*) was used to adjust the pH to 6.8 after the addition of the SIF. Samples (2 mL) were withdrawn from the dissolution media and filtered through a hydrophilic 0.45 µm filter (Millipore, Millex-LCR, Merck, Madrid, Spain) at 5, 10, 15, and 30 min and 1, 3, 4, and 24 h. Kaletra tablets containing 200 mg of lopinavir and 50 mg of ritonavir were employed for a comparison. Ritonavir and lopinavir were separated on a Thermo BDS Hypersil C18 reverse-phase column (250 × 4.6 mm, 5 µm). HPLC analysis was undertaken using a Varian Prostar 230 Solvent Delivery Module, a Varian Prostar autosampler 410, and a Varian Prostar 310 UV-visible detector (Varian, CA, USA). Integration of the peaks was performed with a Galaxie Chromatography Data System (Varian, CA, USA). The mobile phase (potassium phosphate buffer: acetonitrile, 50:50, *v*:*v*) was pumped at a flow rate of 1 mL/min, and the sample injection volume was 40 µL. The column temperature was kept at 25 °C, and the detector was set at 245 nm.

The dissolution data obtained were fitted using the following kinetic equations [39,40]: zero-order (Equation (1)), first-order (Equation (2)), Hixson–Crowell (Equation (3)), Korsmeyer–Peppas (Equation (4)), and Higuchi (Equation (5)).

*Q_t_ = Q*_0_*+ K*_0_*t* (1)

*LogQ_t_ = logQ_0_ + K*_1_*t2.303* (2)

*W*_0_^1/3^*− W*^1/3^*_t_ = K_s_t* (3)

*Log(Mt/M∞) = log K_kp_ + nlogt* (4)

*Q = tDC_s_(2C − C_s_)* (5)

where *Q_t_* is the amount of drug dissolved in time *t*; *Q*_0_ is the initial amount of drug in the solution (most times, *Q*_0_ = *0*); *W*_0_ is the initial amount of drug in the tablet; *W_t_* is the remaining amount of drug in the tablet; *Mt/M∞* is the fraction of drug release at time *t*; *D* is the diffusion constant; *C* is the initial drug concentration; *C_s_* is the drug solubility in the matrix medium; *Q* is the amount of drug released per time *t* per unit area; *K*_1_ is the first-order release constant; *K*_0_ is the zero-order release constant; *K_s_* is a constant incorporating the surface–volume relation; *K_KP_* is a constant that describes the structural and geometric characteristics of the drug dosage form; and *n* is the release exponent that describes the drug release mechanism. The *n* has a value of 0.5, 0.45, or 0.43 when the particle shape is a thin film, a cylinder, or a sphere, respectively, which indicates Fickian release controlled by diffusion [41]. Anomalous non-Fickian transport is observed when *n* is between those values and 1 (0.5 < *n* < 1 for thin films, 0.45 < *n* < 1 for cylinders, and 0.43 < *n* < 1 for spheres). Values of *n* = 1 correspond to zero-order release [42]. The choice of release profile that best fits the release data was determined based on the obtained regression coefficient (R^2^) [39,41,43].

#### 4.2.9. Statistical Analysis

A one-way ANOVA test was performed for the dissolution data using Minitab 16 (Minitab Ltd., Coventry, UK), followed by Tukey’s test, considering *p*-values for statistical significance to be below 0.05. Linear regression analysis was carried out using the method of least squares using Microsoft Excel 2010 software (Microsoft Corporation, Redmond, WA, USA). The modeling of dissolution curves was carried out using the mathematical software DDsolver (China Pharmaceutical University, Nanjing, China) [44].

## 5. Conclusions

Currently, available treatments for HIV are not well-adapted to children’s specific needs. Protease inhibitors are recommended for the HIV treatment in children. In this work, we have successfully manufactured 6 mm spherical minitablets containing the protease inhibitors ritonavir and lopinavir by using direct powder extrusion. The combination of 25% protease inhibitor, 51.75% HPMCAS, 22% PEG 4000, and 0.75% magnesium stearate resulted in partially amorphous solid dispersions with a zero-order sustained release profile over 24 h keeping both drugs in solution. This release profile has significant advantages compared to commercial formulations of ritonavir/lopinavir, such as Kaletra, that show a sharp drug precipitation at the intestinal pH.

## Figures and Tables

**Table 1:** Summary of 3D printed minitablets with ritonavir and lopinavir (*n* = 5).

Drug	Tablet	Weight (mg)	Diameter (mm)	Density (g/cm^3^)	Drug Content (%)
Ritonavir	Mean	149.3	6.3	1.13	95.8
	SD	11.4	0.4	0.16	5.0
	CV (%)	7.7	5.8	14.11	5.2
Lopinavir	Mean	158.3	6.6	1.06	97.7
	SD	12.04	0.4	0.13	2.6
	CV (%)	7.6	6.8	12.49	2.3

Key: The coefficient of variation (CV) is calculated as the ratio of the standard deviation and the average mean value of each parameter multiplied by 100. The density was calculated by dividing the weight of each minitablet by the volume that occupies what is considered to be a perfect sphere. The dimensions of the minitablet were measured with a caliper (Cole Parmer, Fisher Scientific, Madrid, Spain). For a tablet size between 80 to 250 mg, according to Pharmacopeia specifications for mass uniformity (Appendix number 2.9.5), no more than two tablets can deviate more than 7.5% of the average weight, and no tablet should deviate more than 15% of the average weight. According to Pharmacopeia specifications (Appendix 2.9.6), all tested tablets should contain between 85–115% drug content to meet specifications [21,22].

**Table 2:** 3D printing settings.

Parameter	Value
Temperature	80 °C
Platform Temperature	80 °C
Fan	25%
Layer Height	0.1 mm
Infill	100%
Travel and Print Speed	50 & 10 mm/s
Infill Type	Linear

## Data Availability

Data is contained within the article.
